# SERPINA3K Plays Antioxidant Roles in Cultured Pterygial Epithelial Cells through Regulating ROS System

**DOI:** 10.1371/journal.pone.0108859

**Published:** 2014-10-08

**Authors:** Chengpeng Zhu, Fangyu Pan, Lianping Ge, Jing Zhou, Longlong Chen, Tong Zhou, Rongrong Zong, Xinye Xiao, Nuo Dong, Maomin Yang, Jian-xing Ma, Zuguo Liu, Yueping Zhou

**Affiliations:** 1 Eye Institute of Xiamen University, Fujian Provincial Key Laboratory of Ophthalmology and Visual Science, Xiamen, Fujian, China; 2 Affiliated Xiamen Eye Center of Xiamen University, Xiamen, Fujian, China; 3 Xiamen Kehong Eye Hospital, Xiamen, Fujian, PR China; 4 Department of Physiology, The University of Oklahoma Health Sciences Center, Oklahoma City, Oklahoma, United States of America; National Cancer Center, Japan

## Abstract

We recently demonstrated that SERPINA3K, a serine proteinase inhibitor, has antioxidant activity in the cornea. Here we investigated the antioxidant effects of SERPINA3K on the pterygial, which is partially caused by oxidative stress in pathogenesis. The head part of primary pterygial tissue was dissected and then cultured in keratinocyte serum-free defined medium (KSFM). The cultured pterygial epithelial cells (PECs) were treated with SERPINA3K. The cell proliferation and migration of PECs were measured and analyzed. Western blot and quantitative real-time polymerase chain reaction (PCR) assay were performed. It showed that SERPINA3K significantly suppressed the cell proliferation of PECs in a concentration-dependent manner, compared with cultured human conjunctival epithelial cells. SERPINA3K also inhibited the cell migration of PECs. Towards its underlying mechanism, SERPINA3K had antioxidant activities on the PECs by significantly inhibiting NADPH oxidase 4 (NOX4), which is an important enzyme of ROS generation, and by elevating the levels of key antioxidant factors of ROS: such as NAD(P)H dehydrogenase (quinone 1) (NQO1), NF-E2–related factor-2 (NRF2) and superoxide dismutases (SOD2). Meanwhile, SERPINA3K down-regulated the key effectors of Wnt signaling pathway: β-catenin, nonphospho-β-catenin, and low-density lipoprotein receptor-related protein 6 (LRP6). We provided novel evidence that SERPINA3K had inhibitory effects on pterygium and SERPINA3K played antioxidant role via regulating the ROS system and antioxidants.

## Introduction

Pterygium is a common ocular surface disease with the characteristics of triangle shape pathologic tissue of fibrovascular neoformation, which originates from conjunctiva, eventually invades cornea and will block the vision in severe cases. Pterygium often happens in the specific geographic regions with strong ultraviolet light, such as, South-East Asia, South-East China, Australia, and so on. Extensive research has been done on the pathogenesis of pterygium. Oxidative stress is considered a major pathogenesis of pterygium, there are other causes, for example, ultraviolet radiation-induced DNA injury, limbal stem cells deficiency (LSCD) [1]–[5] while the mechanism of pterygium is not fully understood. Meanwhile, there is no effective medication to treat pterygium or prevent the development of pterygium, the current main treatment is to remove the pterygium by surgery and the relapse rate after surgery is high [6], [7].

Multiple recent investigations suggest that the epithelial cells of pterygium are highly proliferative, with tumor cell like cell property [8]–[10]. This high cell proliferation leads to the rapid development and high rate of relapse of pterygium in the clinic. It needs better elucidation on the mechanism of pterygium and exploration of new inhibitory agents to hamper the development of pterygium.

SERPINA3K is a member of the family of serine proteinase inhibitors. SERPINA3K is expressed in the liver, kidney, and ocular tissues. SERPINA3K was first identified as a specific inhibitor of tissue kallikrein, also known as kallikrein-binding protein, since it specifically binds with tissue kallikrein to form a covalent complex and inhibits its proteolytic activities [11]. We recently reported that SERPINA3K has antiinflammatory, antiangiogenic and antioxidant activity in the corneal epithelium [12], [13]. SERPINA3K is also believed to be an inhibitor of Wnt signaling pathway [14]. In this present study, we, for the first time, investigated the inhibitory effects of SERPINA3K on the epithelial cells of pterygium and the underlying mechanism by focusing on reactive oxygen species (ROS) system and Wnt signaling pathway.

## Methods

### Patients

Seventy-six primary pterygium patients were recruited, irrespective of sex (18 cases of men and 58 cases of women) and age (25–76 years old, mean of age: 50±3.4). The conjunctiva samples were collected from 10 strabismus patients, irrespective of sex and age (2–18 years old). All cases were carefully diagnosed clinically with routine examinations and slit-lamp observation. The patients were not found any severe ocular complications, for example, corneal ulcer, and so on, when recruited. The patients underwent surgery at Xiamen Eye Center. All investigations were conducted in accordance with the tenets of the Declaration of Helsinki and were approved by the Ethics Committee of Xiamen Eye Center (an affiliated hospital of Xiamen University). A written informed consent was acquired from all participating patients. The head part of the pterygium tissue, that is, the part invading cornea, was excised for the cell culture experiment.

### Materials

The CCK-8 assay kits were purchased from Dojindo (Tokyo, Japan). The antibodies of anti-NOX4, anti-NQO1, anti-NRF2, anti-β-catenin, anti-nonphospho-β-catenin, and anti-LRP-6 were purchased from Abcam (Cambridge, MA). AlexaFluor488-conjugated IgG was purchased from Invitrogen (Carlsbad, CA).

### Purification of SERPINA3K

The SA3K/pET28 construct was introduced into Escherichia coli strain BL21. The purification procedure of SERPINA3K has been previously reported [12]. The purity of recombinant SERPINA3K was examined by SDS PAGE. Endotoxin concentration was monitored by using a limulus amebocyte kit. Activity was checked by MTT assay with HUVEC cells.

### Cell Culture

The culture experiments were performed within two hours after surgical removal of pterygium or collection of conjunctiva samples. Fresh head part of pterygial specimens was dissected and conjunctiva tissue was collected. The dissected specimens were then cut into small pieces (1–2 mm in diameter), washed in keratinocyte serum-free defined medium (KSF-M) (GIBCO, Carlsbad, CA) and placed in a culture dish. The culture dish was placed in a CO_2_-regulated incubator with 5% CO_2_. The KSF-M medium was replaced every 2 days for about 15 days until the appearance of an outgrowth of pterygial or conjunctival epithelial cells. The procedures of pterygium epithelial cell culture and identification were previously described and followed [15].

### Experimental Procedures

First passage of pterygial epithelial cells (PECs) or conjunctival epithelial cells collected from explant culture were cultured and used in the formal experiments until the cells were cultured to 75% confluency for cell viability measurement and 90% confluency for the cell migration test, respectively. SERPINA3K at concentrations given was added in the media for 12 or 24 hours in the treatment groups before experimental measurements and assays.

### Cell Viability

The cultured pterygial epithelial cells (PECs) and conjunctival epithelial cells were used. Cell viability or cell proliferation of PECs and conjunctival epithelial cells was measured by the CCK-8 assay. CCK-8 assay was conducted with the protocol of the manufacturer. Briefly, after incubation in conditional media for 24 hours, the media were replaced by CCK-8 constituted in culture media, followed by incubation for 4 hours at 37°C in the dark. The CCK-8 containing medium was detected directly after incubation. The absorbance was measured spectrophotometrically at 570 nm with a Bio Tek ELX800 microplate reader (Bio-Tek Instruments, Winooski, VT).

### Cell Migration

A scratch wound test was conducted to detect the cell migration of PECs. Briefly when PECs cells were cultured to 90% confluency, a scratch was applied in the center of the culture dish. The cell images were recorded at 0 and 12 hours. The length of the unmigrated or uninvading area was measured and analyzed to represent the cell migration of PECs.

### Western Blot

Total cellular proteins of the harvested PECs cells were extracted. The standard Western blot protocol was applied. The specific primary antibodies of anti-NOX4, anti-NQO1, anti-NRF2, anti-β-catenin, anti-nonphospho-β-catenin, anti-LRP-6, and a horseradish peroxidase–conjugated secondary antibody were used. Finally, the specific bands were visualized by enhanced chemiluminescence reagents and recorded on film.

### Quantitative Real-time Polymerase Chain Reaction (PCR) Assay

Total RNA was extracted from the cultured PECs cells by using TRIzol reagent (Invitrogen, Carlsbad, CA.). Reverse transcription was performed with Oligo 18T primers and reverse transcription reagents according to the manufacturer's protocol (TaKaRa, Shiga, Japan). Quantitative real-time polymerase chain reaction (PCR) was performed with mRNA special primers. The following primers were used for the PCR assay: for NOX4, 5′-TATCCAGTCCTTCCGTTGGTT-3′ (forward) and 5′-CTGAGGTACAGCTGGATGTTGA-3′ (reverse); for SOD2, 5′-GAGAAGTACCAGGAGGCGTTG-3′ (forward) and 5′-GAGCCTTGGACACCAACAGAT-3′ (reverse) and for NRF-2, 5′-AAACCAGTGGATCTGCCAAC-3′(forward) and 5′-GACCGGGAATATCAGGAACA-3′(reverse). PCR reactions were performed on a BIO-RAD CFX-96 Real Time system with SYBR Premix Ex Taq (TaKaRa, Shiga, Japan) at 95°C for 10 minutes, followed by 45 cycles of 95°C for 10 seconds, 57°C for 30 seconds, and 75°C for 10 seconds, after which melt curve analysis was performed at once from 65°C to 95°C. All reactions were performed in triplicate and the average cycle threshold (Ct) values greater than 38 were treated as negative. The level of GAPDH mRNA was used as an internal control.

### Statistical Analysis

One-way analysis of variance test (ANONA) was conducted to analyze the data of CCK-8 assay, scratch wound test, Western blot, and quantitative real-time PCR assay followed by a post hoc analysis Tukey test to compare the differences between the groups or a Student's t-test. A value of p<0.05 was considered statistically significant.

## Results

### SERPINA3K Suppressed Cell Viability and Migration of Pterygium Epithelial Cells

**Figure 1:**
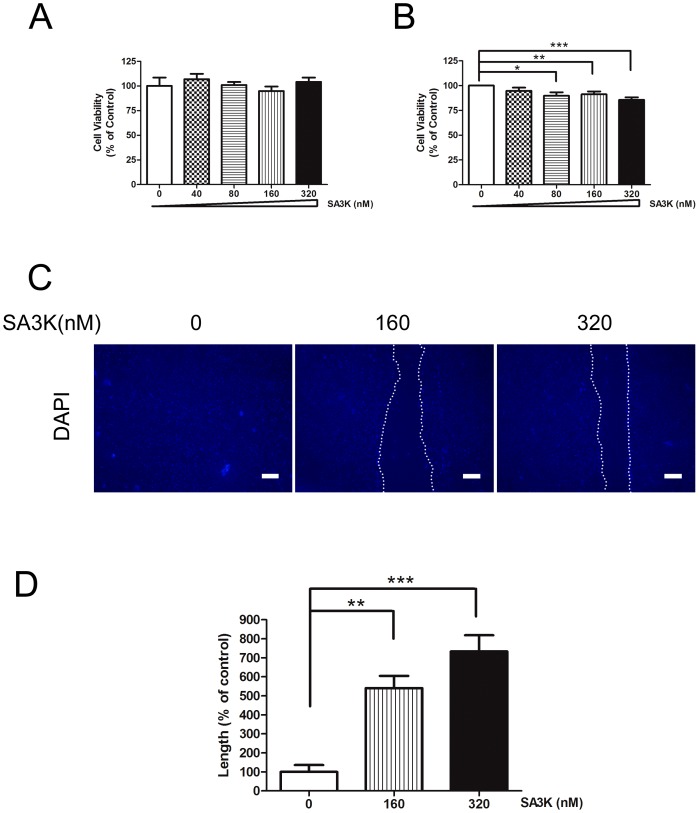
SERPINA3K (SA3K) suppressed the cell proliferation and cell migration of pterygial epithelial cells (PECs). Comparison of the effects of SERPINA3K on the cell proliferation of conjunctival epithelial cells (**A**) and PECs (**B**). Conjunctival epithelial cells and PECs were cultured in keratinocyte serum-free defined medium (KSF-M) and treated with SA3K at concentrations of 0, 40, 80, 160, and 320 nM for 24 hours. Data were presented as mean±SEM; n = 9–10; *: p<0.05, **: p<0.01, ***: p<0.001. (**C**) Representative images of cell migration of PECs after treatment of SA3K (0, 160 and 320 nM) for 12 hours. The central dotted area demonstrated the unmigrated or uninvading area of PECs. Scale bar,100 µm (**D**) Statistic analysis of the length of the unmigrated or uninvading area between control, 160 nM and 320 nM SA3K groups. Data were presented as mean ±SEM; n = 4; **: p<0.01, ***: p<0.001.

Since the epithelial cells of pterygium are highly proliferative [8]–[10], we first evaluated the inhibitory effects of SERPINA3K (SA3K) on the cell viability or cell proliferation of cultured pterygial epithelial cells (PECs) and compared PECs with cultured human conjunctival epithelial cells. It demonstrated that SA3K statistically significantly suppressed the cell proliferation of PECs at concentration of 80, 160 and 320 nM after treatment of 24 hours (Figure 1B). Meanwhile, SA3K at same concentrations did not influence the cell proliferation of cultured human conjunctival epithelial cells significantly (Figure 1A), indicating that SERPINA3K may selectively and specifically suppress the cell proliferation of pterygial epithelial cells. We next investigated the effects of SA3K on the cell migration of cultured PECs using scratch wound test. SA3K at concentration of 160 nM and 320 nM significantly inhibited the cell migration of PECs after treatment of 12 hours. (Figure 1C, 1D) These data indicated that SERPINA3K has inhibitory effects on pterygium.

### SERPINA3K Regulated the ROS Generation Enzyme and Antioxidants

Oxidative stress is the major pathogenesis of pterygium [1]–[5], we recently reported that SERPINA3K plays an antioxidant role in the corneal epithelium [13], we then identified if SERPINA3K inhibited PECs through targeting the reactive oxygen species (ROS) system, for example, regulations of the key enzyme of the ROS generation system: NADPH oxidase 4 (NOX4) [16]–[18] and the antioxidants of the ROS system: such as, NAD(P)H dehydrogenase (quinone 1) (NQO1), NF-E2–related factor-2 (NRF2) and superoxide dismutases (SOD2) [19]–[23].

**Figure 2:**
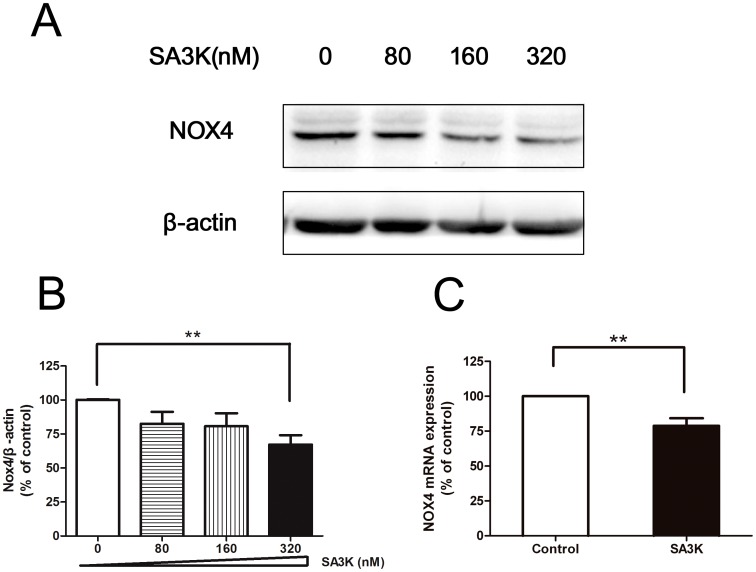
SERPINA3K (SA3K) inhibited the key ROS generation enzyme: NOX4. (**A**) Representative images of Western blot with anti-NOX4 antibody in the PECs after treatment of SA3K at concentration of 0, 80, 160, and 320 nM for 24 hours. (**B**) Statistic analysis of the Western blot data. Data were presented as mean±SEM; n = 5; **: p<0.01. (**C**) Quantitative real-time PCR assay data of NOX4 in the PECs after treatment of 320 nM SA3K for 24 hours. Data were presented as mean±SEM; n = 5; **: p<0.01.

We first examined the effect of SERPINA3K on the ROS generation enzyme NOX4 by Western blot with specific anti-NOX4 antibody. It showed that SA3K significantly downregulated the protein level of NOX4 after treatment of 24 hours (Figure 2A, 2B). In addition, we also detected the gene expression of NOX4 by quantitative real-time PCR assay. SA3K significantly inhibited the gene expression of NOX4 in the PECs after treatment of 24 hours. (Figure 2C).

**Figure 3:**
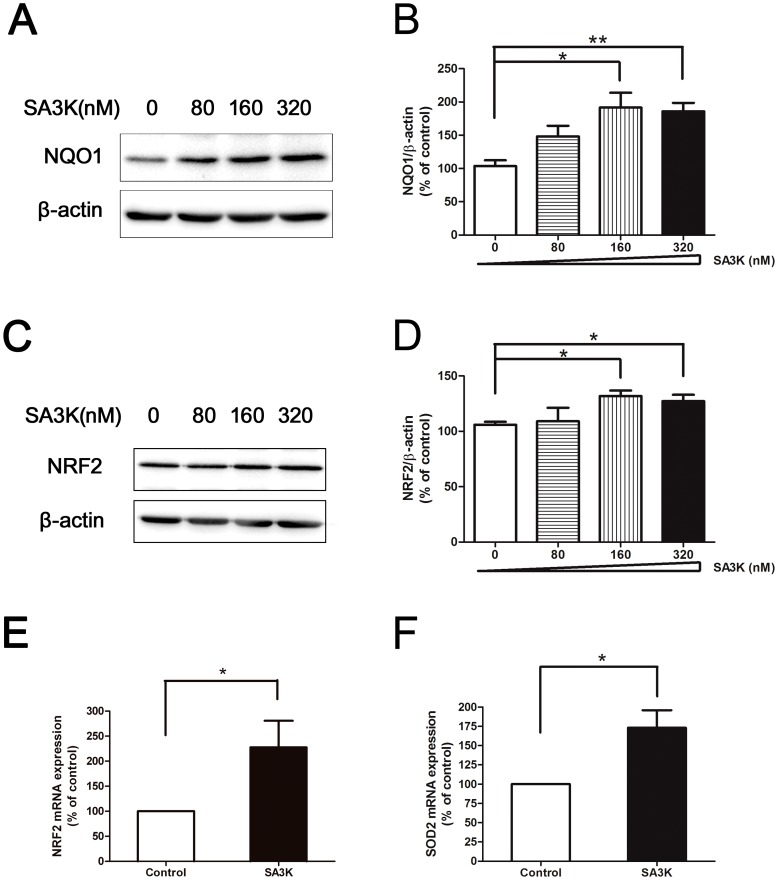
SERPINA3K (SA3K) increased the levels of antioxidants: NQO1, NRF2 and SOD2. (**A**) Representative images of Western blot with anti-NQO1 antibody in the PECs after treatment of SA3K at concentration of 0, 80, 160, and 320 nM for 24 hours. (**B**) Statistic analysis of Western blot data of NQO1. Data were presented as mean±SEM; n = 3; *: p<0.05, **: p<0.01. (**C**) Representative images of Western blot with anti-NRF2 antibody in the PECs after treatment of SA3K at concentration of 0, 80, 160 and 320 nM for 24 hours. (**D**) Statistic analysis of the western blot data of NRF2. Data were presented as mean±SEM; n = 3; *: p<0.05. (**E**) Quantitative real-time PCR assay data of NRF2 in the PECs after treatment of 320 nM SA3K for 24 hours. Data were presented as mean±SEM; n = 6; *: p<0.05. (**F**) Quantitative real-time PCR assay data of SOD2 in the PECs after treatment of 320 nM SA3K for 24 hours. Data were presented as mean±SEM; n = 3; *: p<0.05.

On the other hand, we evaluated the effects of SA3K on the antioxidants of ROS system: NQO1, NRF2 and SOD2. It was demonstrated by Western blot that SA3K significantly increased the protein level of NQO1 after treatment of 24 hours (Figure 3A, 3B). We also detected the changes of gene expression and protein level of antioxidant NRF2 in the PECs after treatment of SA3K for 24 hours. The Western blot results revealed that SA3K significantly increased the protein level of NRF2 in the PECs (Figure, 3C, 3D). Furthermore, it was also shown by quantitative real-time PCR assay that the gene expression of NRF2 was significantly increased in the PECs (Figure 3E). Moreover, SA3K also statistically significantly elevated the gene expression of another antioxidant SOD2 in the PECs after treatment of 24 hours (Figure 3F).

These data suggested that SERPINA3K protects against oxidative stress of PECs via blocking the ROS generation enzyme and increasing the antioxidants.

### SERPINA3K Downregulated Wnt Signaling Pathway

**Figure 4:**
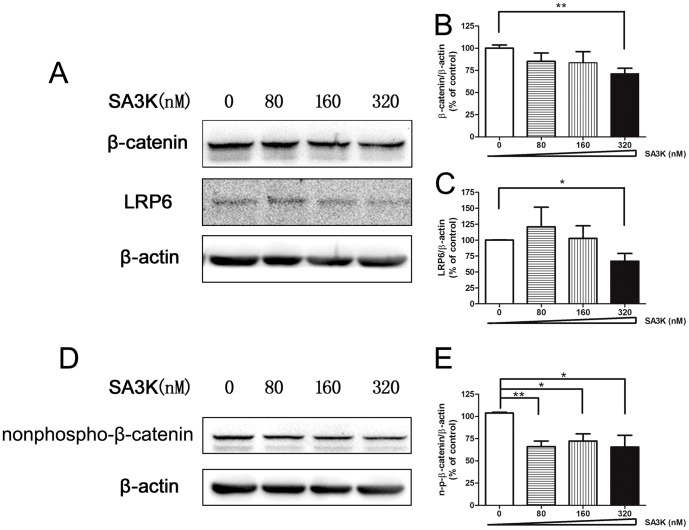
SERPINA3K (SA3K) downregulated Wnt signaling pathway. (**A**) Representative images of Western blot with anti-β-catenin and anti-LRP6 antibodies in the PECs after treatment of SA3K at concentration of 0, 80, 160, and 320 nM for 24 hours. (**B**) Statistic analysis of the western blot data of β-catenin. Data were presented as mean±SEM; n = 4; **: p<0.01. (**C**) Statistic analysis of the Western blot data of LRP6. Data were presented as mean±SEM; n = 5; *: p<0.05. (**D**) Representative images of Western blot with anti-nonphospho-β-catenin antibody in the PECs after treatment of SA3K at concentration of 0, 80, 160 and 320 nM for 24 hours. (**E**) Statistic analysis of the Western blot data of nonphospho-β-catenin. Data were presented as mean±SEM; n = 3; *: p<0.05, **: p<0.01.

We previously demonstrated that SERPINA3K is a Wnt signaling pathway inhibitor via binding and blocking the Wnt pathway upstream effector: low-density lipoprotein receptor-related protein 6 (LRP6) [14], we then determined if the inhibitory effects of SERPINA3K on the PECs are associated with the downregulation of Wnt signaling pathway in the present study. To evaluate the downregulation of Wnt pathway in the PECs, we conducted Western blot of the key effectors of Wnt pathway: β-catenin and LRP6. The Western blot data showed that the protein levels of β-catenin and LRP6 were decreased in the PECs after treatment of SA3K for 24 hours (Figure 4A, 4B, 4C). To further confirm the association of SA3K with the Wnt pathway in the PECs, we also measured another key Wnt pathway effector: nonphospho-β-catenin by Western blot. SA3K at concentration of 80, 160 and 320 nM statistically significantly decreased the protein level of nonphospho-β-catenin after treatment of 24 hours (Figure 4D, 4E). These results suggested that SA3K downregulates the Wnt signaling pathway.

## Discussion

Pterygium is a pathologic ocular surface tissue with highly proliferative cells [8]–[10] and it still lacks of effective medication except the surgical removal. In this study, we, for the first time, demonstrated that SERPINA3K, a serine proteinase inhibitor, suppresses the cell proliferation and migration of pterygial epithelial cells. Moreover, we also revealed that the underlying mechanism of SERPINA3K is through regulations of ROS system and the blockade of Wnt signaling pathway. Our experimental data may contribute to the exploration of new therapeutic agents to antagonize the formation and development of pterygium.

SERPINA3K has been demonstrated to have antiinflammation and antioxidatant activities in the eyes including corneas and retina [12], [13], [24], [25]. It has not been reported about the effect of SERPINA3K on the pterygium, which is associated with oxidative stress and inflammation in pathogenesis. The experimental evidence from this study supported our previous findings of antiinflammatory and antioxidant effects of SERPINA3K in another ocular pathologic tissue and status. It suggested that SERAPINA3K, as an endogenous protein with multiple functions including antiinflammatory, antiangiogenic and antioxidant effects, may have an advantage to treat pterygium and prevent the development of pterygium compared with the current application of antiinflammatory medication after surgical removal of pterygium.

A pterygium originates from conjunctiva and it invades cornea in many cases, it is necessary to determine if SERPINA3K selectively and specifically targets the pterygial epithelial cells, by comparison of the effects of SERPINA3K on conjunctival epithelial cells and corneal epithelial cells. It was revealed by the present investigation that SERPINA3K did not suppress the cell proliferation of cultured human conjunctival epithelial cells. In addition, we previously demonstrated that SERPINA3K at various concentrations did not inhibit the cell proliferation of human corneal epithelial cells (HCEs) [12]. These data suggest that SERPINA3K can specifically suppress the cell proliferation of pterygial epithelial cells. On the other hand, as a pterygium is composed of epithelial layer and the basal layer, which contains lots of fibroblasts or stroma cells, we just demonstrated that SERPINA3K has inhibitory effects on the cell proliferation and migration of epithelial cells of pterygium in this study, it needs further investigation to elucidate the effect of SERPINA3K on pterygial fibroblasts or stroma cells.

Due to the limitation of application of SERPINA3K on human pterygium patients in clinic and the lack of good in vivo animal models of pterygium at present time, we only performed the investigation of SERPINA3K in the cultured pterygial cells, a further investigation on the efficacy of local application of SERPINA3K on animal model of pterygium in vivo and human pterygium should be conducted when available. Furthermore, we mainly focused on the effect of SERPINA3K in primary pterygium in this study, it is of worth to elucidate the effect of SERPINA3K on recurrent pterygium in the future.

Oxidative stress is believed to play a vital role in the pathogenesis and development of pterygium [1]–[5]. Oxidative stress is also a major pathogenesis of other eye diseases, for example, other ocular surface diseases, age-related eye diseases, and so on. [28]–[30] Oxidative stress is a pathologic state of excessive ROS production or abnormal balance of the ROS system or ROS pathway [31], [32]. In this study, we demonstrated that SERPINA3K suppresses the generation enzyme of ROS: NOX4, which is a key enzyme of ROS generation [16]–[18], meanwhile, SERPINA3K also elevated the levels of various antioxidant factors, such as NQO1, NRF2 and SOD2 [19]–[23]. This present study is consistent with our previous report about the antioxidant activity of SERPINA3K in the corneal epithelium [13]. Since we mainly focused on the antioxidant role of SERPINA3K in pterygium, it is necessary to make a further comparison of the antioxidant activities of SERPINA3K between the pterygium, conjunctiva and corneas with same range and stages of age to better understand and evaluate the antioxidant role of SERPINA3K in the formation and development of pterygium.

It is reported that SERPINA3K is an inhibitor of Wnt signaling pathway, the target and binding site of SERPINA3K is the upstream effector of Wnt pathway: LRP6 [14]. Thus we evaluated the alteration of Wnt pathway after treatment of SERPINA3K in the cultured pterygial epithelial cells, we demonstrated that SERPINA3K down-regulated the Wnt pathway, including the effectors: LRP6, β-catenin and nonphospho-β-catenin. This result also supports the hypothesis that SERPINA3K is an inhibitor of Wnt signaling pathway in different ocular tissue and cells, while the more detailed molecular mechanistic needs further elucidated, for example, how SERPINA3K affects the activities of downstream factors of Wnt pathway in the pterygial epithelial cells, such as transcription factor 4 (TCF4), and so on. It also requires a better understanding of the role of Wnt pathway in the formation and development of pterygium and to compare the effects of SERPINA3K on Wnt pathway between pterygium, conjunctiva as well as cornea, while there are multiply reports indicating that Wnt signaling pathway is highly expressed or activated in the pterygium [26], [27].

Taken together, we provided novel experimental evidence that SERPINA3K may inhibit the formation and development of pterygium and SERPINA3K has antioxidant activity in pterygium, indicating that SERPINA3K is of the potential to be used as a therapeutic agent in the treatment of pterygium or to prevent the relapse of pterygium after surgical removal in clinic.
